# Finerenone use and incident psoriasis in patients with type 2 diabetes and chronic kidney disease: A retrospective cohort study using the TriNetX network

**DOI:** 10.1016/j.jdin.2026.06.010

**Published:** 2026-06-22

**Authors:** Wu-Lung Chuang, Ruey-Hwang Chou, Yuan-Hung Wang, I-Ju Tsai, Cheng-Li Lin, Der-Yang Cho, Kuang-Hsi Chang

**Affiliations:** a Division of Endocrinology and Metabolism, Department of Internal Medicine, Changhua Christian Hospital, Changhua, Taiwan; b Division of Endocrinology and Metabolism, Department of Internal Medicine, Lukang Christian Hospital, Changhua, Taiwan; c Graduate Institute of Biomedical Sciences, China Medical University, Taichung, Taiwan; d Center for Molecular Medicine, China Medical University Hospital, Taichung, Taiwan; e Department of Medical Laboratory and Biotechnology, Asia University, Taichung, Taiwan; f Graduate Institute of Clinical Medicine, College of Medicine, Taipei Medical University, Taipei, Taiwan; g Department of Medical Research, Shuang Ho Hospital, Taipei Medical University, New Taipei City, Taiwan; h Management Office for Health Data, China Medical University Hospital, Taichung, Taiwan; i College of Medicine, China Medical University, Taichung, Taiwan; j Department of Medical Research, Translational Cell Therapy Center, China Medical University Hospital, Taichung, Taiwan; k Department of Neurosurgery, China Medical University Hospital, Taichung, Taiwan; l Graduate Institute of Biomedical Sciences, China Medical University, Taichung, Taiwan; m Department of Long-Term Care, College of Nursing, Asia University, Taichung, Taiwan

**Keywords:** chronic kidney disease, epidemiology, finerenone, psoriasis, real-world evidence, type 2 diabetes mellitus

*To the Editor:* Psoriasis is a chronic immune-mediated disease involving tumor necrosis factor, interleukin (IL)-23, and IL-17.^1^ Psoriasis is associated with systemic comorbidities, including metabolic and renal diseases.^2^ Finerenone improves kidney and cardiovascular outcomes in patients with type 2 diabetes mellitus (T2DM) and chronic kidney disease (CKD),^3^^,^^4^ and its anti-inflammatory profile suggests biologic plausibility for psoriasis risk.^5^ However, clinical evidence on this question remains limited.

We performed a retrospective cohort study using the TriNetX Global Research Network. Adults with newly diagnosed T2DM after July 2021 who subsequently developed CKD were included. Patients with prior psoriasis, prior finerenone exposure, prior sodium-glucose cotransporter 2 inhibitor use, CKD preceding T2DM, age younger than 18 years, or missing sex data were excluded. The primary outcome was new-onset psoriasis, defined as the first ICD-10 code L40 recorded after the index date. Patients were followed until psoriasis diagnosis, death, loss to follow-up, or the end of the observation period, whichever occurred first. Baseline covariates included age, sex, and major comorbidities identified from ICD-10 codes, including hypertension (HT: I10-I15), dyslipidemia (DL: E78), heart failure (HF: I50), atrial fibrillation/flutter (AF/AFL: I48), acute myocardial infarction (AMI: I21), stroke (I63, I61), obesity (E66), chronic obstructive pulmonary disease (COPD: J44), asthma (J45-J46), Chronic liver disease (CLD: K70, K73, K74), and neoplasms (C00-D49); these variables were used for propensity score matching and baseline comparison.

Among 67,995 eligible patients, 1717 finerenone users were matched 1:1 to 1717 nonusers with balanced baseline characteristics. Because the outcome analysis was restricted to incident psoriasis, patients with a documented history of psoriasis before the index date were excluded from the outcome-specific risk set. Therefore, the numbers of patients at risk in Table I were slightly lower than the original matched cohort. During follow-up, psoriasis occurred in 13 finerenone users and 63 nonusers. Finerenone use was associated with a lower risk of incident psoriasis (adjusted hazard ratio, 0.19; 95% CI, 0.11-0.35). Kaplan-Meier analysis showed a significantly higher psoriasis-free probability in the finerenone group (Figure 1).

**Table I:** Association between finerenone use and the risk of psoriasis among patients with diabetic kidney disease

	*N*	Event	aHR (95% CI)
Non-Finerenone	1675	63	1.00
Finerenone	1710	13	0.19 (0.11, 0.35)

Event: number of patients with psoriasis; aHR: adjusted hazard ratio, adjusted by age, sex, HT, DL, HF, AF/AFL, AMI, stroke, obesity, COPD, asthma, CLD, and neoplasms.

**Fig 1:**
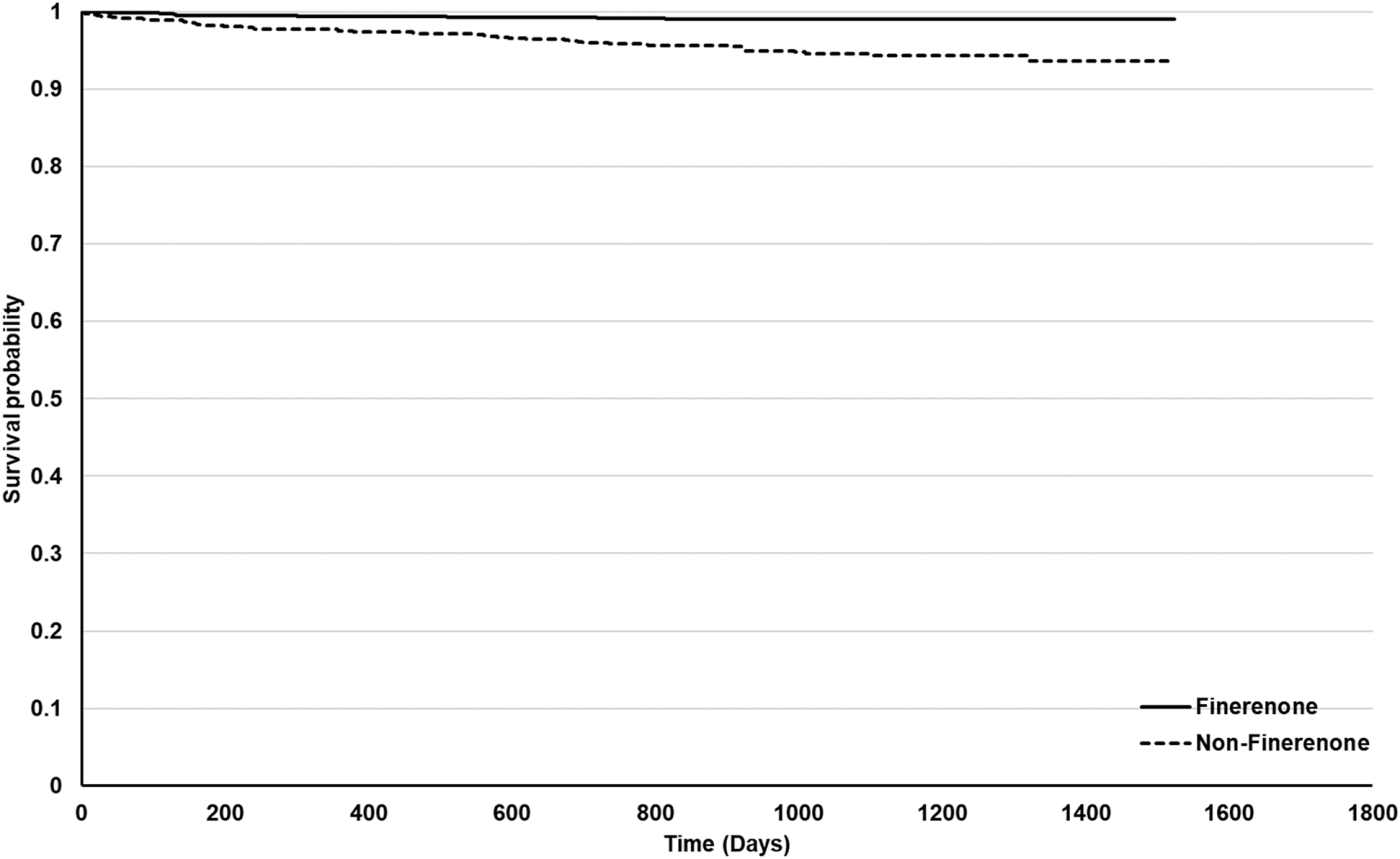
Kaplan-Meier survival curves for psoriasis-free probability in finerenone users versus nonusers.

These findings should be interpreted cautiously because the small number of psoriasis events makes them hypothesis-generating rather than definitive. Residual confounding by disease severity, adherence, body mass index, smoking, race/ethnicity and lifestyle and prescribing factors can’t be excluded. Excluding prior SGLT2 inhibitor users may have reduced generalizability and introduced selection bias. Exposure and outcome were identified from electronic health record coding and prescription data, and incident psoriasis was defined using ICD-10 code L40 without independent validation. Laboratory data, psoriasis severity measures, and detailed treatment duration were unavailable. Accordingly, the findings should be interpreted as an association rather than a causal effect.

In summary, finerenone use was associated with a lower incidence of psoriasis in this multinational real-world cohort of patients with T2DM and CKD; however, given the limited events, observational design, and potential multiplicity, this finding should not be interpreted as evidence of a protective effect. Further prospective and mechanistic studies are warranted to validate this association.

## Conflicts of interest

None disclosed.
